# Loss of base-to-apex circumferential strain gradient assessed by cardiovascular magnetic resonance in Fabry disease: relationship to T1 mapping, late gadolinium enhancement and hypertrophy

**DOI:** 10.1186/s12968-019-0557-0

**Published:** 2019-08-01

**Authors:** Shobhit Mathur, John G. Dreisbach, Gauri R. Karur, Robert M. Iwanochko, Chantal F. Morel, Syed Wasim, Elsie T. Nguyen, Bernd J. Wintersperger, Kate Hanneman

**Affiliations:** 1 Toronto Joint Department of Medical Imaging, Toronto General Hospital, University of Toronto, 585 University Avenue, 1PMB-298, Toronto, ON M5G 2N2 Canada; 2 Division of Cardiology, Peter Munk Cardiac Centre, University Health Network, University of Toronto, 585 University Ave, Toronto, ON M5G 2N2 Canada; 3 Fred A. Litwin Centre in Genetic Medicine, University Health Network & Mount Sinai Hospital, University of Toronto, 60 Murray St, Toronto, ON M5T 3L9 Canada

**Keywords:** Cardiomyopathy, Fabry disease, Cardiovascular magnetic resonance, Magnetic resonance imaging, T1 mapping, Myocardial strain

## Abstract

**Background:**

Cardiac involvement is common and is the leading cause of mortality in Fabry disease (FD). We explored the association between cardiovascular magnetic resonance (CMR) myocardial strain, T1 mapping, late gadolinium enhancement (LGE) and left ventricular hypertrophy (LVH) in patients with FD.

**Methods:**

In this prospective study, 38 FD patients (45.0 ± 14.5 years, 37% male) and 8 healthy controls (40.1 ± 13.7 years, 63% male) underwent 3 T CMR including cine balanced steady-state free precession (bSSFP), LGE and modified Look-Locker Inversion recovery (MOLLI) T1 mapping. Global longitudinal (GLS) and circumferential (GCS) strain and base-to-apex longitudinal strain (LS) and circumferential strain (CS) gradients were derived from cine bSSFP images using feature tracking analysis.

**Results:**

Among FD patients, 8 had LVH (FD LVH+, 21%) and 17 had LGE (FD LGE+, 45%). Nineteen FD patients (50%) had neither LVH nor LGE (FD LVH- LGE-). None of the healthy controls had LVH or LGE. FD patients and healthy controls did not differ significantly with respect to GLS (− 15.3 ± 3.5% vs. − 16.3 ± 1.5%, *p* = 0.45), GCS (− 19.4 ± 3.0% vs. -19.5 ± 2.9%, *p* = 0.84) or base-to-apex LS gradient (7.5 ± 3.8% vs. 9.3 ± 3.5%, *p* = 0.24). FD patients had significantly lower base-to-apex CS gradient (2.1 ± 3.7% vs. 6.5 ± 2.2%, *p* = 0.002) and native T1 (1170.2 ± 37.5 ms vs. 1239.0 ± 18.0 ms, *p* < 0.001). Base-to-apex CS gradient differentiated FD LVH- LGE- patients from healthy controls (OR 0.42, 95% CI: 0.20 to 0.86, *p* = 0.019), even after controlling for native T1 (OR 0.24, 95% CI: 0.06 to 0.99, *p* = 0.049). In a nested logistic regression model with native T1, model fit was significantly improved by the addition of base-to-apex CS gradient (χ^2^(df = 1) = 11.04, *p* < 0.001). Intra- and inter-observer agreement were moderate to good for myocardial strain parameters: GLS (ICC 0.849 and 0.774, respectively), GCS (ICC 0.831 and 0.833, respectively), and base-to-apex CS gradient (ICC 0.737 and 0.613, respectively).

**Conclusions:**

CMR reproducibly identifies myocardial strain abnormalities in FD. Loss of base-to-apex CS gradient may be an early marker of cardiac involvement in FD, with independent and incremental value beyond native T1.

## Background

Fabry disease (FD) is an X-linked disorder characterized by progressive accumulation of lysosomal sphingolipids in multiple organs [1]. Cardiac involvement is common and is the leading cause of mortality in patients with FD [2–4].

Cardiovascular magnetic resonance (CMR) is routinely used in the evaluation of patients with FD, including assessment of left ventricular hypertrophy (LVH), late gadolinium enhancement (LGE) and native T1 mapping [5–7]. CMR findings have an important clinical role in FD, affecting prognosis and treatment decisions [8, 9].

Myocardial strain abnormalities assessed by echocardiography have been described in FD, including reductions in longitudinal (LS) and circumferential strain (CS) and loss of base-to-apex CS gradient [10–13]. However, there are limited data on CMR derived myocardial strain in FD, particularly in relation to other CMR parameters such as native T1. Feature tracking strain analysis of cine CMR images allows for quantification of myocardial deformation without the need to acquire additional dedicated sequences, and has been shown to predict adverse clinical outcomes in other cardiomyopathies [14, 15]. Detection of myocardial strain abnormalities with CMR could potentially allow for earlier identification of cardiac involvement in FD.

We sought to explore the association between CMR feature tracking derived myocardial strain, LVH, LGE and T1 mapping in FD patients. We hypothesize that CMR identified loss of base-to-apex CS gradient may be an early marker of cardiac involvement in FD.

## Methods

### Study population

From June 2016 to May 2018, consecutive FD patients undergoing 3 T CMR at our institution were prospectively enrolled. The study was approved by the institutional research ethics board and written informed consent was obtained from all subjects. Inclusion criteria for patients included age ≥ 18 years and confirmed diagnosis of FD. Exclusion criteria included prior myocardial infarction or known coronary artery disease and contraindications to CMR. A reference healthy control group was also imaged, defined as volunteers without past medical history of cardiovascular disease. The final study population consisted of thirty-eight gene-positive FD patients (mean age 45.0 ± 14.5 years, 37% male) and eight healthy controls (mean age 40.1 ± 13.7 years, 63% male).

### CMR technique

All CMR studies were performed using a 3 T scanner (Magnetom Skyra Fit, Siemens Healthineers, Erlangen, Germany or Biograph mMR, Siemens Healthineers). Multi-planar retrospectively gated cine balanced steady-state free precession (bSSFP) images were obtained including multiple long-axis planes and a stack of short-axis slices with coverage from cardiac base to apex (8 mm slice thickness and 2 mm inter-slice gap). Multi-planar LGE imaging was performed approximately 15 min after administration of 0.15 mmol/kg body weight of gadobutrol (Gadovist, Bayer Healthcare, Berlin, Germany) employing a phase sensitive inversion recovery gradient-recalled echo sequence (8 mm slice thickness and 2 mm inter-slice gap) at matching slice positions. Native and 12–15 min post-contrast mid-ventricular short-axis T1 maps were acquired using a bSSFP readout modified Look-Locker inversion recovery (MOLLI) technique with inline motion correction and map creation (native (5(3)3) and post-contrast (4(1)3(1)2) inversion groupings, 8 mm slice thickness) [16].

### CMR analysis

**Fig. 1:**
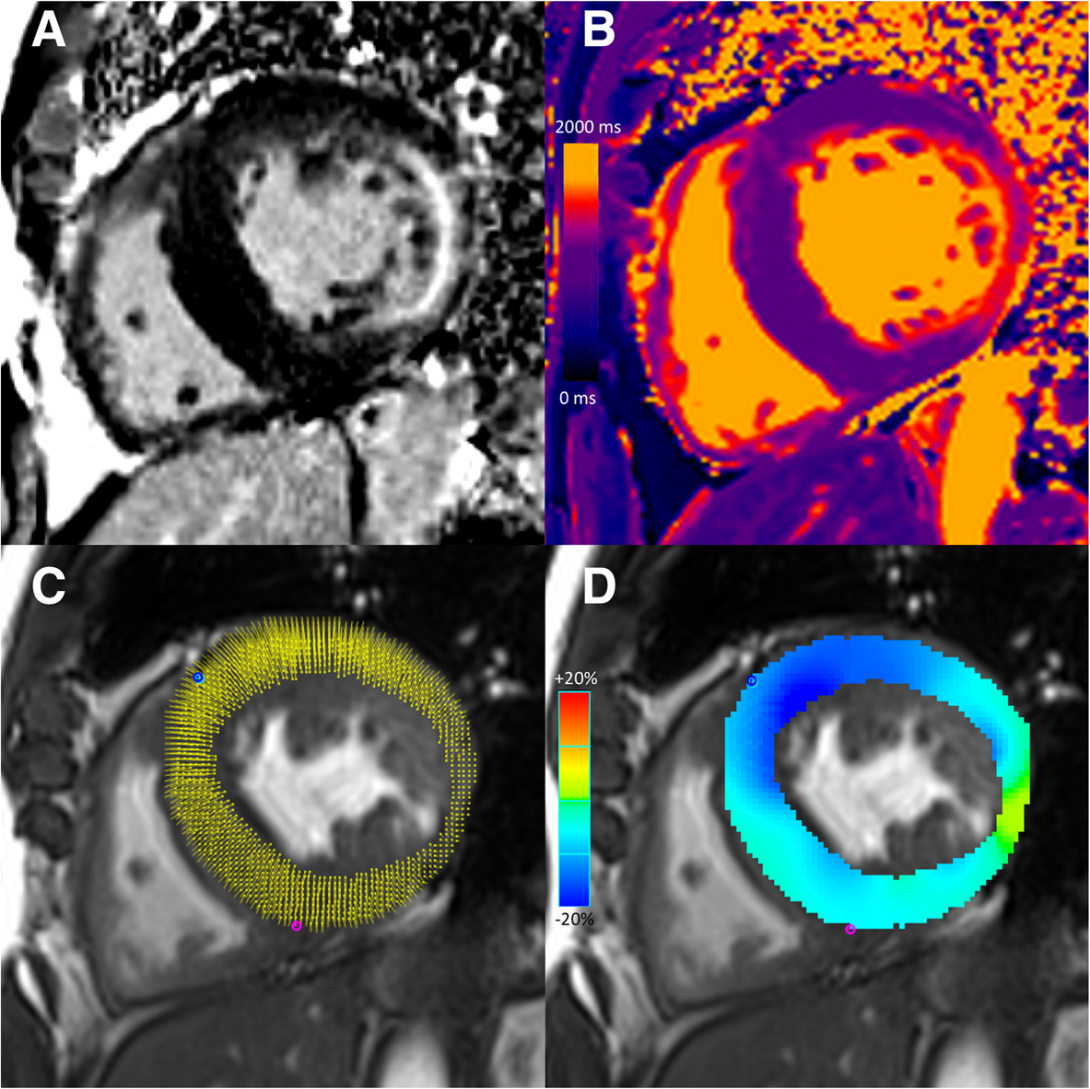
Cardiovascular magnetic resonance images of a 57-year-old male with Fabry disease. Short-axis late gadolinium enhanced (LGE) image demonstrates concentric left ventricular hypertrophy and mid-wall LGE at the inferior lateral wall (**a)**. Short-axis native T1 map (native T1 value 1100 ms at the interventricular septum) (**b**). Short-axis cine balanced steady state free precession (bSSFP) image with circumferential myocardial strain analysis points (**c**) and color myocardial circumferential strain map (**d**). Global longitudinal strain was − 10.4%. Global circumferential strain was − 15.0%. Base-to-apex longitudinal strain gradient was 2.6%. Base-to-apex circumferential strain gradient was 2.1%

CMR analysis was performed by a fellowship-trained radiologist (SM, with 2 years of cardiovascular imaging experience) blinded to all identifying information using commercially available software (Circle cmr42 release 5.6.3; Circle Cardiovascular Imaging, Calgary, Canada). LV endocardial and epicardial borders were contoured on the stack of short-axis bSSFP images to assess for left ventricular (LV) volumes, LV ejection fraction (LVEF), and mass, and on short axis LGE images to assess for the extent of LGE. LVH was defined as LV mass indexed to body surface area (BSA) > 85 g/m^2^ in males and > 81 g/m^2^ in females [17]. All LGE images were visually assessed for the presence of LGE. Quantitative LGE analysis was performed using a signal intensity threshold of 4 standard deviations above visually normal remote myocardium, expressed as a percentage of total myocardial mass [18]. For feature tracking strain analysis, endocardial and epicardial borders were contoured at end-diastole on short-axis (at basal, mid-ventricle and apical levels) and long-axis (2-, 3- and 4-chamber) bSSFP images. Peak myocardial global longitudinal strain (GLS) was calculated by averaging all peak longitudinal myocardial values. Peak global CS (GCS) was obtained by averaging peak myocardial strain values across basal, mid and apical levels (Fig. 1). Base-to-apex LS and CS gradients were calculated as the peak gradient difference between basal and apical LS and CS, respectively [12]. T1 values were evaluated by drawing a single region of interest in the mid interventricular septum excluding any areas with LGE [6, 19]. Myocardial extracellular volume (ECV) was calculated with input of native and post-contrast myocardial and blood pool T1 values, and hematocrit obtained within 24 h of CMR by non-invasive testing [20, 21].

### Intra- and inter-observer agreement

To assess intra-observer agreement for strain analysis, a random subset of 15 studies were re-analyzed by the same reader following a minimum 2-week interval after the first analysis, blinded to the results of the initial assessment and all identifying data. To assess inter-observer agreement, the same subset of studies was analyzed by a second experienced fellowship-trained reader (JD, with 2 years of cardiovascular imaging experience), blinded to all identifying information including the results of the initial assessment.

### Statistical analysis

Statistical analysis was performed using STATA v14.1 (StataCorp, College Station, Texas, USA). A two-tailed *p*-value of < 0.05 was considered statistically significant. Continuous variables were described using mean and standard deviation and categorical variables using numbers and percentage. All continuous data were tested for normal distribution using the Shapiro-Wilk test. Comparisons between groups were made by independent samples t-test for continuous variables with normal distribution, Wilcoxon rank-sum test for continuous variables with non-normal distribution, and Fisher’s exact test for categorical variables. Correlation between continuous variables was assessed using Spearman correlation. Univariable logistic regression was used to evaluate the ability of native T1, ECV, GLS, GCS, base-to-apex LS gradient and base-to-apex CS gradient to differentiate FD patients without LVH or LGE from healthy controls. To evaluate whether sex is an effect modifier, interaction terms for sex and base-to-apex CS gradient and for sex and native T1 were included in respective logistic regression models along with sex as a main effect and tested for significance. A bivariable logistic regression model was fitted with base-to-apex CS gradient and native T1. For assessment of incremental diagnostic value, the likelihood ratio test was used to compare nested models with native T1 versus one with base-to-apex CS gradient added. Intra- and inter-observer agreement were assessed using individual intra-class correlation coefficients with one-way random effects models and two-way random effects models, respectively.

## Results

Baseline clinical information is summarized in Table 1. CMR findings are summarized in Table 2. None of the healthy controls had LVH or LGE. Among FD patients, 8 had LVH (FD LVH+, 21%) and 17 had LGE (FD LGE+, 45%). Nineteen FD patients (50%) had neither LVH nor LGE (FD LVH- LGE-).

**Table 1:** Baseline characteristics

	Healthy Controls (*n* = 8)	Fabry Disease Patients (*n* = 38)	*p*-value
Age (years)	40.1 ± 13.7	45.0 ± 14.5	0.39
Female	3 (38%)	24 (63%)	0.25
Male	5 (63%)	14 (37%)	0.25
Hematocrit (%)	41 ± 5	41 ± 4	0.96
Height (cm)	168.0 ± 14.0	166.5 ± 9.3	0.71
Weight (kg)	72.3 ± 15.1	75.2 ± 15.8	0.64
BSA (m^2^)	1.83 ± 0.25	1.85 ± 0.21	0.81
Systolic blood pressure (mmHg)		120.6 ± 15.1	
Diastolic blood rpessure (mmHg)		76.9 ± 9.5	
Hypertension		13 (34%)	
Diabetes mellitus		2 (5%)	
Chronic kidney disease		0 (0%)	
Medications			
Enzyme replacement therapy		13 (34%)	
Beta blockers		6 (16%)	
Statins		17 (45%)	
Calcium channel blocker		4 (11%)	
ACEi/ARB		15 (40%)	
Aspirin		15 (40%)	

Comparisons between groups were made by independent samples t-test, Wilcoxon rank sum test or Fisher’s exact test. Continuous variables are presented as mean ± SD. Categorical variables are presented as number of subjects with percentage in parentheses. *ACEi* angiotensin converting enzyme inhibitor, *ARB* angiotensin II receptor blocker, *BSA* body surface area

**Table 2:** Cardiovascular magnetic resonance findings

	Healthy Controls (*n* = 8)	Fabry Disease Patients (*n* = 38)	*p*-value
LVEDV (mL)	178.5 ± 46.4	154.6 ± 34.9	0.16
LVEDVi (mL/m^2^)	96.3 ± 13.9	84.2 ± 20.2	0.043
LVESV (mL)	73.1 ± 23.8	63.2 ± 18.1	0.23
LVESVi (mL/m^2^)	39.3 ± 8.7	34.4 ± 10.3	0.10
LVSV (mL)	105.4 ± 23.3	91.4 ± 20.0	0.09
LVEF (%)	59.6 ± 3.6	59.31 ± 4.6	0.86
Cardiac output (L/min)	6.3 ± 1.5	6.2 ± 1.2	0.86
LV mass (g)	99.9 ± 37.1	131.2 ± 57.8	0.22
LVMi (g/m^2^)	53.4 ± 15.7	71.0 ± 31.5	0.21
LGE (presence)	0.0 (0%)	17 (45%)	0.019
LGE (%)	0.0 ± 0.0	2.0 ± 3.5	< 0.001
Native T1 (ms)	1239.0 ± 18.0	1170.2 ± 37.5	< 0.001
ECV (%)	25.9 ± 3.2	25.6 ± 2.9	0.48
GLS (%)	-16.3 ± 1.5	−15.3 ± 3.5	0.45
GCS (%)	−19.5 ± 2.9	−19.4 ± 3.0	0.84
Base-to-apex LS gradient (%)	9.3 ± 3.5	7.5 ± 3.8	0.24
Base-to-apex CS gradient (%)	6.5 ± 2.2	2.1 ± 3.7	0.002

Comparisons between groups were made by independent samples t-test, Wilcoxon rank sum test or Fisher’s exact test. Continuous variables are presented as mean ± SD. Categorical variables are presented as number of subjects with percentage in parentheses. *CS* circumferential strain, *ECV* extracellular volume, *FD* Fabry disease, *GCS* global circumferential strain, *GLS* global longitudinal strain, *LGE* late gadolinium enhancement, *LS* longitudinal strain, *LV* Left ventricle, *LVMi* left ventricular mass indexed to body surface area (BSA), *LVEF* left ventricular ejection fraction, *LVEDV* left ventricular end diastolic volume, *LVEDVi* LVEDV indexed to BSA, *LVESV* left ventricular end systolic volume, *LVESVi* LVESV indexed to BSA, *LVSV* left ventricular stroke volume

### T1 mapping

**Fig. 2:**
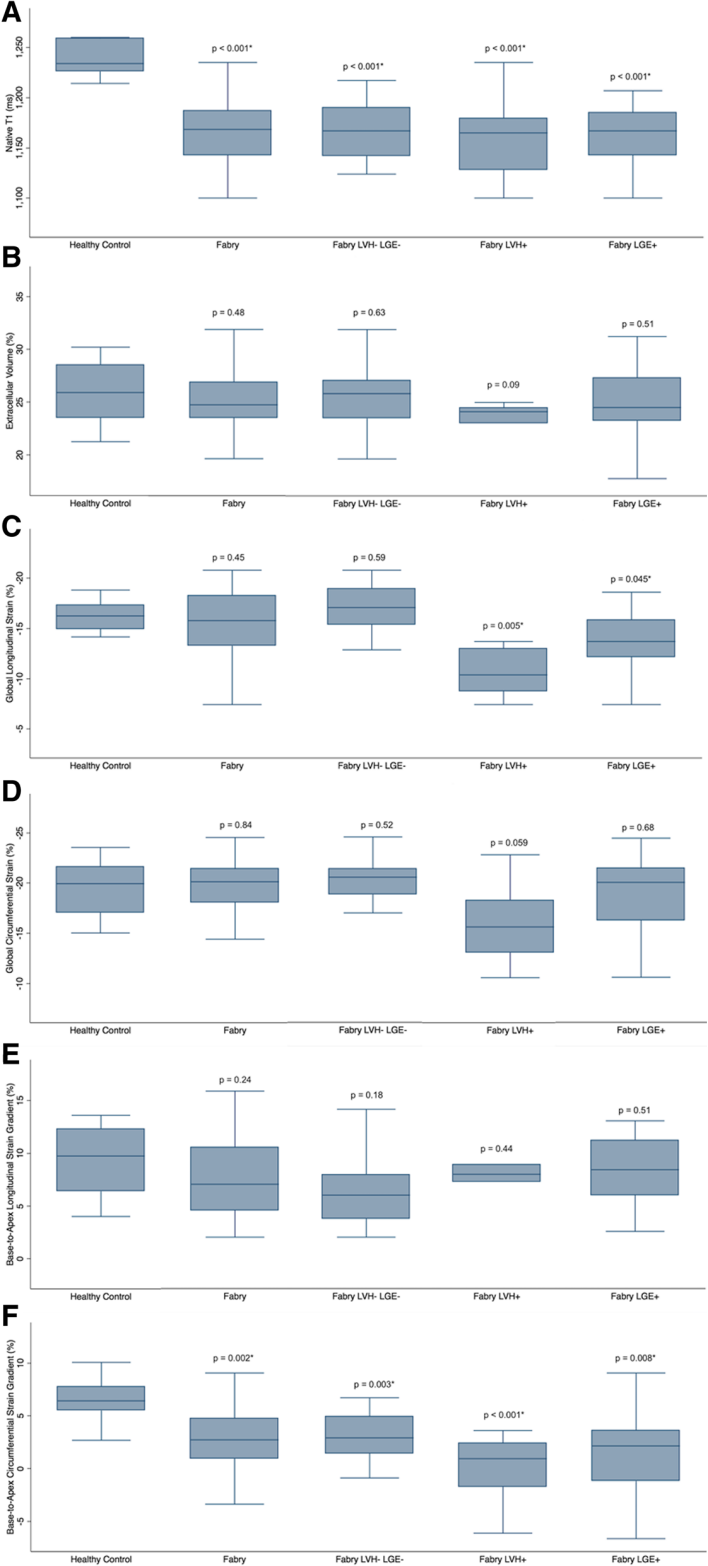
Box plots comparing T1 mapping and strain values between healthy controls and Fabry disease patients and different sub-groups: all Fabry disease (Fabry) patients, Fabry patients without left ventricular hypertrophy (LVH) or late gadolinium enhancement (LGE) (LVH- LGE-), Fabry patients with LVH (LVH+) and Fabry patients with LGE (LGE+). All *p*-values are for comparisons between healthy controls and Fabry patient groups. Native T1 (**a**). Extracellular volume fraction (**b**). Global longitudinal strain (**c**). Global circumferential strain (**d**). Base-to-apex longitudinal strain gradient (**e**). Base-to-apex circumferential strain gradient (**f**)

Native T1 values were significantly lower in FD patients compared to controls (1170.2 ± 37.5 vs. 1239.0 ± 18.0 ms, *p* < 0.001), even when the comparison was restricted to the LVH- LGE- subgroup (1172.2 ± 42.4 ms, *p* < 0.001), FD LVH+ subgroup (1160.1 ± 41.6 ms, *p* < 0.001), and FD LGE+ subgroup (1163.2 ± 29.9 ms, *p* < 0.001) (Fig. 2a). There was no significant difference in ECV between FD patients and controls (25.1 ± 2.9% vs. 25.9 ± 3.2%, *p* = 0.48), even when the comparison was restricted to the FD LVH- LGE- subgroup (25.3 ± 2.8%, *p* = 0.63), FD LVH+ subgroup (23.2 ± 2.5%, *p* = 0.09), and FD LGE + subgroup (24.9 ± 3.4%, *p* = 0.51) (Fig. 2b). Among FD patients, native T1 and ECV were both significantly lower in males compared to females (1154.6 ± 34.8 vs. 1179.3 ± 36.6 ms, *p* = 0.049 and 23.4 ± 2.1% vs. 26.0 ± 3.0%, *p* = 0.009).

### Global longitudinal strain

There was no significant difference in GLS between FD patients and controls (− 15.3 ± 3.5% vs. -16.3 ± 1.5%, *p* = 0.45), even when the comparison was restricted to the FD LVH- LGE- subgroup (− 16.8 ± 2.8%, *p* = 0.59) (Fig. 2c). However, GLS was significantly lower in the FD LVH+ subgroup (− 11.4 ± 3.9%, *p* = 0.005) and FD LGE + subgroup (− 13.6 ± 3.3%, *p* = 0.045) when compared to controls. Among patients with FD, GLS correlated positively with LV mass indexed to BSA (r = 0.64, *p* = 0.02) and quantitative LGE (r = 0.508, *p* = 0.002), but did not correlate significantly with native T1 (r = 0.112, *p* = 0.51) or ECV (r = − 0.206, *p* = 0.23), Table 3. Among FD patients, there was no significant difference in GLS between males and females (− 14.0 ± 4.1% vs. -16.0 ± 2.9%, *p* = 0.09).

**Table 3:** Correlation of myocardial strain values with mass, late gadolinium enhancement and T1 mapping values among patients with Fabry disease

	Global Longitudinal Strain (GLS)		Global Circumferential Strain (GCS)		Base-to-apex Longitudinal Strain Gradient		Base-to-apex Circumferential Strain Gradient	
	r-value	*p*-value	r-value	*p*-value	r-value	*p*-value	r-value	*p*-value
LVMi (g/m^2^)	0.375	0.02	0.426	0.01	−0.101	0.60	−0.255	0.13
LGE (%)	0.508	0.002	0.298	0.08	−0.097	0.61	−0.089	0.61
Native T1 (ms)	0.112	0.51	−0.085	0.62	−0.006	0.98	−0.243	0.15
ECV (%)	−0.206	0.23	−0.250	0.14	−0.071	0.71	−0.265	0.12

Correlation was assessed using Spearman correlation. *ECV* extracellular volume, *GCS* global circumferential strain, *GLS* global longitudinal strain, *LGE* late gadolinium enhancement, *LVMi* left ventricular mass indexed to body surface area

### Global circumferential strain

There was no significant difference in GCS between FD patients and controls (− 19.4 ± 3.0% vs. -19.5 ± 2.9%, *p* = 0.84), even when the comparison was restricted to the FD LVH- LGE- subgroup (− 20.2 ± 1.8% *p* = 0.52) and the FD LGE+ subgroup (− 18.4 ± 3.8%, *p* = 0.68) (Fig. 2d). The difference in GCS between the FD LVH+ subgroup (− 15.9 ± 3.9%) and controls approached statistical significance, *p* = 0.059. Among patients with FD, GCS correlated positively with LV mass indexed to body surface area (BSA) (r = 0.426, *p* = 0.01), but did not correlate significantly with quantitative LGE (r = 0.298, *p* = 0.08), native T1 (r = − 0.085, *p* = 0.62), or ECV (r = − 0.250, *p* = 0.14). Among FD patients, GCS was significantly lower in males compared to females (− 17.8 ± 3.4% vs. -20.4 ± 2.3%, *p* = 0.009).

### Base-to-apex longitudinal strain gradient

There was no significant difference in base-to-apex LS gradient between FD patients and controls (7.5 ± 3.8% vs. 9.3 ± 3.5%, *p* = 0.24), even when the comparison was restricted to the FD LVH- LGE- subgroup (6.9 ± 4.1%, *p* = 0.18), FD LVH+ subgroup (7.7 ± 3.4%, *p* = 0.44), and FD LGE+ subgroup (8.3 ± 3.4%, *p* = 0.51) (Fig. 2e). Among patients with FD, base-to-apex LS gradient did not correlate significantly with LV mass indexed to BSA (r = − 0.101, *p* = 0.60), quantitative LGE (r = − 0.097, *p* = 0.61), native T1 (r = − 0.006, *p* = 0.98) or ECV (r = − 0.071, *p* = 0.71). Among FD patients, there was no significant difference in base-to-apex LS gradient between males and females (7.5 ± 3.7% vs. 7.6 ± 3.9%, *p* = 0.98).

### Base-to-apex circumferential strain gradient

Base-to-apex CS gradient was significantly lower in FD patients compared to controls (2.1 ± 3.7% vs. 6.5 ± 2.2%, *p* = 0.002), even when the comparison was restricted to the LVH- LGE- subgroup (2.7 ± 3.0%, *p* = 0.003), FD LVH+ subgroup (0.1 ± 3.2%, *p* < 0.001), and FD LGE+ subgroup (1.7 ± 4.4%, *p* = 0.008) (Fig. 2f). Among patients with FD, base-to-apex CS gradient did not correlate significantly with LV mass indexed to BSA (r = − 0.255, *p* = 0.13), quantitative LGE (r = − 0.089, *p* = 0.61), native T1 (r = − 0.243, *p* = 0.15) or ECV (r = − 0.265, *p* = 0.12). Among FD patients, there was no significant difference in base-to-apex CS gradient between males and females (2.2 ± 3.5% vs. 2.0 ± 3.9%, *p* = 0.93).

### Discrimination between FD LVH- LGE- patients and controls

Base-to-apex CS gradient (odds ratio [OR] 0.42, 95% confidence interval [CI]: 0.20 to 0.86, *p* = 0.019) and native T1 (OR 0.96, 95% CI: 0.92 to 0.99, *p* = 0.010) were significant in differentiating FD LVH- LGE- patients from healthy controls in univariable models, Table 4. There was no evidence that sex was a modifying variable of the relationship between base-to-apex CS gradient (interaction term, *p* = 0.41) or native T1 (interaction term, *p* = 0.99) and diagnosis of FD. Base-to-apex CS gradient remained significant in a bivariable logistic regression model after adjusting for native T1 (OR 0.24, 95% CI: 0.06 to 0.99, *p* = 0.049). In this bivariable model, native T1 approached but did not reach statistical significance (OR 0.94, 95% CI: 0.88 to 1.00, *p* = 0.056). In a nested logistic regression model with native T1, model fit was significantly improved by the addition of base-to-apex CS gradient (χ^2^(df = 1) = 11.04, *p* < 0.001).

**Table 4:** Univariable logistic regression for CMR parameters to differentiate patients with Fabry disease without left ventricular hypertrophy or late gadolinium enhancement from healthy controls

	Odds Ratio (OR)	95% Confidence Interval	*p*-value
Native T1 (ms)	0.96	0.92, 0.99	0.010
ECV (%)	0.93	0.69, 1.25	0.62
GLS (%)	0.91	0.64, 1.27	0.57
GCS (%)	0.85	0.57, 1.27	0.43
Base-to-apex LS gradient (%)	0.86	0.69, 1.07	0.18
Base-to-apex CS gradient (%)	0.42	0.20, 0.86	0.019

Univariable logistic regression results evaluating cardiovascular magnetic resonance parameters to differential patients with Fabry disease without left ventricular hypertrophy or late gadolinium enhancement from healthy controls. *CS* circumferential strain, *ECV* extracellular volume, *GCS* global circumferential strain, *GLS* global longitudinal strain, *LS* longitudinal strain

### Enzyme replacement therapy

Among patients with FD, 13 (34%) were treated with enzyme replacement therapy. There was no significant difference in native T1, ECV, GLS, GCS, base-to-apex LS gradient or base-to-apex CS gradient between FD patients treated with enzyme replacement therapy and those that were not on therapy (*p* > 0.05 for all).

### Intra- and inter-observer agreement

Intra- and inter-observer agreement were moderate to good for myocardial strain parameters: GLS (ICC 0.849 and 0.774, respectively), GCS (ICC 0.831 and 0.833, respectively), and base-to-apex CS gradient (ICC 0.737 and 0.613, respectively).

## Discussion

The results of this study demonstrate that base-to-apex CS gradient discriminates between FD patients without hypertrophy or LGE and healthy controls independent of native T1, suggesting that loss of base-to-apex CS gradient may be an early marker of cardiac involvement in FD.

A few prior studies have evaluated global strain abnormalities using echocardiography in patients with FD, demonstrating minor reductions in GLS and GCS [10, 11]. Gruner et al. found that GLS and GCS were reduced in FD patients with and without LVH, although there was a greater decrease in GLS among LVH positive patients [10]. Loss of base-to-apex CS gradient assessed by echocardiography has previously been reported in patients with FD [10, 12]. The current study confirms this finding using CMR feature tracking strain analysis, and provides additional information on the association of base-to-apex CS gradient with other CMR parameters including T1 mapping.

To our knowledge, only a few prior studies have reported CMR strain results in patients with FD, and none have included assessment of base-to-apex CS gradient. Cheng-Baron et al. assessed myocardial strain in FD using an alternative method, global fractional shortening, and reported that patients with FD had significant differences in strain parameters compared to healthy controls [22]. Rutz et al. assessed myocardial strain using CMR tissue tagging and harmonic phase analysis (HARP), and found reduced peak values for GLS and GCS in FD patients with LVH, but not in those without LVH [23]. We found that GCS was not significantly reduced in patients with FD and that GLS was only significantly reduced in LVH+ or LGE+ FD patients, suggesting that aberrations in global strain may be relatively late signs of cardiac involvement in FD.

CS and LS normally increase from LV base to apex, which could be due to differences in wall thickness, regional myocardial architecture and local stress [24, 25]. The underlying pathophysiological mechanism resulting in loss of base-to-apex CS gradient in FD has not yet been established, but could relate to the effect of myocardial hypertrophy and fibrosis on contractility. FD cardiomyopathy predominantly involves the mid-myocardial wall, which mostly contributes to circumferential contraction and strain [26]. In other non-ischemic cardiomyopathies, mid-wall fibrosis has been shown to impair circumferential strain [27].

Other patterns of abnormal regional myocardial strain have been described in other cardiomyopathies. For example, a pronounced base-to-apex LS gradient with preserved apical longitudinal strain has been shown to differentiate cardiac amyloid from other causes of LVH including FD [28, 29]. We found that there was no significant difference in base-to-apex LS gradient between FD patients and controls, similar to a prior study that evaluated strain using echocardiography [12].

Early detection of cardiac involvement in FD is critical given the availability of disease specific treatment with intravenous infusion of the deficient enzyme or oral pharmacologic chaperone therapy. Recent studies indicate that low native T1 may be an early biomarker of cardiac involvement in FD, even before the development of LVH [6, 30, 31]. The results of our study demonstrate that loss of base-to-apex CS gradient may be another early marker of cardiac involvement in FD, with independent and incremental diagnostic value beyond native T1 in LVH- and LGE- FD patients. These findings provide further evidence to support a pre-hypertrophic FD phenotype, which could potentially inform guidelines recommending earlier initiation of disease specific treatment [31].

Normal myocardial strain values have been shown to vary between studies depending on the imaging acquisition technique, analysis method and post-processing vendor. Absolute GLS and GCS values for healthy controls in our study are slightly lower compared to prior studies, potentially related to the post-processing vendor used [32, 33]. Absolute myocardial strain values assessed with speckle-tracking echocardiography have also been shown to vary depending on the post-processing vendor used, even when identical loops are analyzed, likely due to differences in tracking algorithms [34, 35]. Given known differences in absolute strain values depending on several factors, we have not provided a cut-point for strain parameters to distinguish between FD patients and controls, although this could be explored in a future study.

There are several limitations to our study. FD is a relatively rare disease, thus limiting the number of subjects included. Adjustment for covariates in logistic regression models is limited by the number of subjects. CMR imaging was only performed at 3 T and all analysis was performed using a single post-processing vendor, and therefore results may not be generalizable to different field strengths or software analysis. Given the small number of subjects, we were not able to adjust our analysis for the duration of therapy or underlying disease severity. Future studies including larger numbers of subjects should be performed to investigate whether loss of base-to-apex CS gradient is specific for cardiac involvement in FD, to evaluate the effect of treatment on base-to-apex CS gradient as an imaging end-point, and to assess the prognostic significance of this finding.

## Conclusions

CMR reproducibly identifies myocardial strain abnormalities in FD. Loss of base-to-apex CS gradient may be an early marker of cardiac involvement in FD, with independent and incremental value beyond native T1.

## Data Availability

The datasets used and/or analysed during the current study are available from the corresponding author on reasonable request.

## Abbreviations

BSA: Body surface area
bSSFP: Balanced steady state free precession
CMR: Cardiovascular magnetic resonance
CS: Circumferential strain
ECV: Extracellular volume
FD: Fabry disease
GCS: Global circumferential strain
GLS: Global longitudinal strain
ICC: Intra-class correlation coefficient
LGE: Late gadolinium enhancement
LS: Longitudinal strain
LV: Left ventricle/left ventricular
LVEF: Left ventricular ejection fraction
LVH: Left ventricular hypertrophy
MOLLI: Modified Look-Locker Inversion

## References

[1] Clarke JTR (2007). Narrative review: Fabry disease. Ann Intern Med.

[2] Patel MR, Cecchi F, Cizmarik M, Kantola I, Linhart A, Nicholls K (2011). Cardiovascular events in patients with fabry disease natural history data from the fabry registry. J Am Coll Cardiol.

[3] Wilson HC, Hopkin RJ, Madueme PC, Czosek RJ, Bailey LA, Taylor MD (2017). Arrhythmia and clinical cardiac findings in children with Anderson-Fabry disease. Am J Cardiol.

[4] Linhart A, Kampmann C, Zamorano JL, Sunder-Plassmann G, Beck M, Mehta A (2007). Cardiac manifestations of Anderson-Fabry disease: results from the international Fabry outcome survey. Eur Heart J.

[5] Deva DP, Hanneman K, Li Q, Ng MY, Wasim S, Morel C (2016). Cardiovascular magnetic resonance demonstration of the spectrum of morphological phenotypes and patterns of myocardial scarring in Anderson-Fabry disease. J Cardiovasc Magn Reson.

[6] Karur GR, Robison S, Iwanochko RM, Morel CF, Crean AM, Thavendiranathan P (2018). Use of myocardial T1 mapping at 3.0 T to differentiate Anderson-Fabry disease from hypertrophic cardiomyopathy. Radiology.

[7] Kozor R, Grieve SM, Tchan MC, Callaghan F, Hamilton-Craig C, Denaro C (2016). Cardiac involvement in genotype-positive Fabry disease patients assessed by cardiovascular MR. Heart.

[8] Hanneman K, Karur GR, Wasim S, Morel CF, Iwanochko RM (2018). Prognostic significance of cardiac magnetic resonance imaging late gadolinium enhancement in Fabry disease. Circulation.

[9] Arends M, Biegstraaten M, Hughes DA, Mehta A, Elliott PM, Oder D (2017). Retrospective study of long-term outcomes of enzyme replacement therapy in Fabry disease: analysis of prognostic factors. PLoS One.

[10] Gruner C, Verocai F, Carasso S, Vannan MA, Jamorski M, Clarke JTR (2012). Systolic myocardial mechanics in patients with Anderson-Fabry disease with and without left ventricular hypertrophy and in comparison to nonobstructive hypertrophic cardiomyopathy. Echocardiography.

[11] Shanks M, Thompson RB, Paterson ID, Putko B, Khan A, Chan A (2013). Systolic and diastolic function assessment in fabry disease patients using speckle-tracking imaging and comparison with conventional echocardiographic measurements. J Am Soc Echocardiogr.

[12] Labombarda F, Saloux E, Milesi G, Bienvenu B (2017). Loss of base-to-apex circumferential strain gradient: a specific pattern of Fabry cardiomyopathy?. Echocardiograph.

[13] Krämer J, Niemann M, Liu D, Hu K, Machann W, Beer M (2013). Two-dimensional speckle tracking as a non-invasive tool for identification of myocardial fibrosis in Fabry disease. Eur Heart J.

[14] Eitel I, Stiermaier T, Lange T, Rommel K-P, Koschalka A, Kowallick JT (2018). Cardiac magnetic resonance myocardial feature tracking for optimized prediction of cardiovascular events following myocardial infarction. JACC Cardiovasc Imaging.

[15] Smith BM, Dorfman AL, Yu S, Russell MW, Agarwal PP, Ghadimi Mahani M (2014). Relation of strain by feature tracking and clinical outcome in children, adolescents, and young adults with hypertrophic cardiomyopathy. Am J Cardiol.

[16] Kellman P, Arai AE, Xue H (2013). T1 and extracellular volume mapping in the heart: estimation of error maps and the influence of noise on precision. J Cardiovasc Magn Reson.

[17] Kawel-Boehm N, Maceira A, Valsangiacomo Buechel ER, Vogel-Claussen J, Turkbey EB, Williams R (2015). Normal values for cardiovascular magnetic resonance in adults and children. J Cardiovasc Magn Reson.

[18] Moravsky G, Ofek E, Rakowski H, Butany J, Williams L, Ralph-Edwards A (2013). Myocardial fibrosis in hypertrophic cardiomyopathy: accurate reflection of histopathological findings by CMR. JACC Cardiovasc Imaging.

[19] Hanneman K, Crean AM, Wintersperger BJ, Thavendiranathan P, Nguyen ET, Kayedpour C (2018). The relationship between cardiovascular magnetic resonance imaging measurement of extracellular volume fraction and clinical outcomes in adults with repaired tetralogy of Fallot. Eur Heart J Cardiovasc Imaging.

[20] Arheden H, Saeed M, Higgins CB, Gao D-W, Bremerich J, Wyttenbach R (1999). Measurement of the distribution volume of Gadopentetate Dimeglumine at Echo-planar MR imaging to quantify myocardial infarction: comparison with 99mTc-DTPA autoradiography in rats. Radiology.

[21] Robison S, Karur GR, Wald RM, Thavendiranathan P, Crean AM, Hanneman K (2018). Noninvasive hematocrit assessment for cardiovascular magnetic resonance extracellular volume quantification using a point-of-care device and synthetic derivation. J Cardiovasc Magn Reson.

[22] Cheng-Baron J, Chow K, Pagano JJ, Punithakumar K, Paterson DI, Oudit GY (2015). Quantification of circumferential, longitudinal, and radial global fractional shortening using steady-state free precession cines: a comparison with tissue-tracking strain and application in Fabry disease. Magn Reson Med.

[23] Rutz AK, Juli CF, Ryf S, Widmer U, Kozerke S, Eckhardt BP (2007). Altered myocardial motion pattern in Fabry patients assessed with CMR-tagging. J Cardiovasc Magn Reson.

[24] Bogaert J, Rademakers FE (2001). Regional nonuniformity of normal adult human left ventricle. Am J Physiol Heart Circ Physiol.

[25] Nagata Y, VC-C W, Otsuji Y, Takeuchi M (2017). Normal range of myocardial layer-specific strain using two-dimensional speckle tracking echocardiography. Galderisi M, editor. PLoS One.

[26] Scatteia A, Baritussio A, Bucciarelli-Ducci C (2017). Strain imaging using cardiac magnetic resonance. Heart Fail Rev.

[27] Taylor RJ, Umar F, Lin ELS, Ahmed A, Moody WE, Mazur W (2016). Mechanical effects of left ventricular midwall fibrosis in non-ischemic cardiomyopathy. J Cardiovasc Magn Reson.

[28] Liu D, Hu K, Niemann M, Herrmann S, Cikes M, Stork S (2013). Effect of combined systolic and diastolic functional parameter assessment for differentiation of cardiac amyloidosis from other causes of concentric left ventricular hypertrophy. Circ Cardiovasc Imaging..

[29] Phelan D, Collier P, Thavendiranathan P, Popovic ZB, Hanna M, Plana JC (2012). Relative apical sparing of longitudinal strain using two-dimensional speckle-tracking echocardiography is both sensitive and specific for the diagnosis of cardiac amyloidosis. Heart.

[30] Pica S, Sado DM, Maestrini V, Fontana M, White SK, Treibel T (2014). Reproducibility of native myocardial T1 mapping in the assessment of Fabry disease and its role in early detection of cardiac involvement by cardiovascular magnetic resonance. J Cardiovasc Magn Reson.

[31] Nordin S, Kozor R, Baig S, Abdel-Gadir A, Medina-Menacho K, Rosmini S (2018). Cardiac phenotype of Prehypertrophic Fabry disease. Circ Cardiovasc Imaging.

[32] Vo HQ, Marwick TH, Negishi K (2018). MRI-derived myocardial strain measures in Normal subjects. JACC Cardiovasc Imaging.

[33] Schuster A, Stahnke V-C, Unterberg-Buchwald C, Kowallick JT, Lamata P, Steinmetz M (2015). Cardiovascular magnetic resonance feature-tracking assessment of myocardial mechanics: Intervendor agreement and considerations regarding reproducibility. Clin Radiol.

[34] Biaggi P, Carasso S, Garceau P, Greutmann M, Gruner C, Tsang W (2011). Comparison of two different speckle tracking software systems: does the method matter?. Echocardiography.

[35] Amzulescu MS, Langet H, Saloux E, Manrique A, Boileau L, Slimani A (2017). Head-to-head comparison of global and regional two-dimensional speckle tracking strain versus cardiac magnetic resonance tagging in a multicenter validation study. Circ Cardiovasc Imaging.

